# Andes virus outbreak linked to expedition cruise ship travel, multi-country investigation and response, April to June 2026

**DOI:** 10.2807/1560-7917.ES.2026.31.24.2600477

**Published:** 2026-06-18

**Authors:** Oda Everdina van den Berg, UKHSA ANDV Team, Ettore Severi, Freddy Mutoka-Banza, Michele van Vugt, Esther M Schadd, María Cruz Calvo Reyes, Laura Santos Larrégola, Pedro Valdivia Prieto, François-Xavier Lescure, Mark A Cachia, Mayank Singal, Mirko Faber, Walter Zingg, Nazir Ahmed Ismail, Margreet JM te Wierik, Tjalling Leenstra, Chantal Reusken, Susan van den Hof, the International ANDV team, UKHSA ANDV Team, Richard Puleston, Petra Manley, Grace Oswald, Will Welfare, Carol Chatt, Liam Fitzpatrick, Meera Chand, Matt Dryden, Jacob Asplin, Thomas Inns, Natalie Wright, Hilary Kirkbride, Richard Pebody, Colin Brown, Anna Aryee, Claire Gordon, Tommy Rampling, Paul Crook, Sherine Thomas, The International ANDV team, Leonidas Alexakis, Agoritsa Baka, Orlando Cenciarelli, Kostas Danis, Luna Eunkyung Park, Céline M Gossner, Andreas Hoefer, Esther Kukielka Zunzunegui, Favelle Lamb, Emma Löf, Paula Luikens, Raffaella Melilli, Despina Pampaka, David Peres, Emmanuel Robesyn, Adriana Romaní Vidal, Gianfranco Spiteri, Giorgia Solda, Maike Winters, Réka Gustafson, Alexandra Septfons, Alexandra Mailles, Arnaud Tarantola, Yvan Souares, Harold Noël, Jean-Claude Manuguerra, Virginie Sauvage, Daniel Bourquain, Raskit Lachmann, Lucía García-San Miguel Rodríguez-Alarcón, Berta Suárez Rodríguez, Bernardo Guzmán Herrador, Esteban Aznar, Sonia Fernández-Balbuena, Alejandro Ciriano Cervantes, Héctor Sanchez Herrero, Juan Antonio del Castillo Polo, Gabriela Saravia Campelli, Fernando Márquez Vita, María de Salomón Arroyo, Pilar Gallego Berciano, María José Sierra Moros, Fernando Simón Soria, Patricia López Pereira, Rocío Palmera-Suárez, Rocío Ruiz Huertas, Beatriz Nieto Pereda, Miguel Dávila-Cornejo, Fernando Riesco Rodriguez, María Paz Sánchez-Seco Fariñas, Ana Isabel Negredo Antón, Ana Vázquez González, Francisco Javier Membrillo de Novales, Tatiana Mata Forte, Ana Boned-Ombuena, Maria Beso Delgado, Paula Silvestre Molines, Katja Villatoro Bongiorno, Esteve Fernández, Jacobo Mendioroz, Laura Esteban, Núria Balanza, Olivia Luise Gern, Mart Stein, Roan Pijnacker, Annelies Brilman, Menno de Jong, Corine GeurtsvanKessel, Annemiek van der Eijk, Lucille Blumberg, Nevashan Govender, Lerato Sikhosana, Jacqueline Weyer, Vuyiswa Kumalo, Emanuele Bruni, Silviu Ciobanu, Ana Paula Coutinho Rehse, Esther Hamblion, Ana Hoxha, Kareena Hundal, Zyleen Kassamali, Karen Nahapetyan, Boris I Pavlin, Ihor Perehinets, Tanja Schmidt, Marc-Alain Widdowson, Dubravka Selenic Minet

**Affiliations:** 1 Centre for Infectious Disease Control, National Institute for Public Health and the Environment (RIVM), Bilthoven, The Netherlands; 2 European Programme for Intervention Epidemiology Training (EPIET), European Centre for Disease Prevention and Control (ECDC), Stockholm, Sweden; 3 The members of the UKHSA ANDV Team are listed under Collaborators; 4 European Centre for Disease Prevention and Control (ECDC), Stockholm, Sweden; 5 World Health Organization Regional Office for Africa (WHO AFRO), Emergency Preparedness and Response, Emergency Response, Brazzaville, Congo; 6 Amsterdam University Medical Centers, Amsterdam, The Netherlands; 7 Department of Internal Medicine, Central Military Hospital, Utrecht, The Netherlands; 8 Coordinating Centre for Health Alerts and Emergencies (CCAES), Ministry of Health, Madrid, Spain; 9 Infectious and Tropical Diseases Department and French National Mission for Epidemic and Biological Risk Coordination, Assistance Publique - Hôpitaux de Paris (APHP), Bichat Hospital and Université Paris Cité, French National Institute of Health and Medical Research (INSERM), Infection, Antimicrobials, Modelling, Evolution (IAME), Paris, France; 10 Population & Public Health, Island Health, Victoria, British Columbia, Canada; 11 British Columbia Centre for Disease Control, Vancouver, British Columbia, Canada; 12 Robert Koch Institute (RKI), Berlin, Germany; 13 Department of Infectious Diseases and Hospital Epidemiology, University Hospital Zurich and University of Zurich, Zurich, Switzerland; 14 The National Institute for Communicable Diseases (NICD), Johannesburg, South Africa; 15 The members of the International ANDV team are listed under Collaborators

**Keywords:** hantavirus, Andes virus, cruise ship, outbreak investigation, repatriation, contact tracing, isolation, quarantine, public health response, laboratory surveillance

## Abstract

As at 9 July 2026^††^, 13 cases (12 confirmed and one probable) of Andes orthohantavirus have been reported (case fatality: 23%), linked to the Dutch-flagged expedition cruise ship m/v *Hondius*. The event involved individuals from 23 nationalities and required medical evacuation, repatriation, coordinated international contact tracing, isolation, quarantine and clinical and laboratory testing follow-up. To date, all cases have been passengers (10/121; 8%) or crew members (3/61; 5%). Ongoing monitoring and investigations aim to clarify the source of the outbreak, identify risk factors and prevent further spread.

A multi-country outbreak of Andes hantavirus among passengers and crew of a Dutch-flagged expedition cruise ship in April to June 2026 including 13 cases as of mid-June, required coordinated international control activities. We describe the public health response and related investigations.

## Outbreak detection and initial investigation

On 2 May 2026, authorities from the United Kingdom (UK) and the Netherlands notified the World Health Organization (WHO) and the European Centre for Disease Prevention and Control (ECDC) of a cluster of severe respiratory illness of unknown aetiology among the passengers and crew onboard the Dutch-flagged expedition cruise ship m/v *Hondius*, which had departed from Ushuaia, Argentina, on 1 April 2026 with destination to Praia, Cape Verde [1]. During April, the ship sailed to South Georgia, Cooper Island, Tristan da Cunha, Gough Island, Saint Helena and Ascension Island, reaching Praia on 3 May [1]. The timelines for the first 10 cases of disease have been described elsewhere [2,3]. Initial testing detected hantavirus. Subsequent laboratory analyses in South Africa and Switzerland confirmed the causal agent as a member of the *Orthohantavirus andesense* complex of viruses (ANDV).

As at 9 July 2026^††^, 13 cases linked with the cruise ship have been reported (12 laboratory-confirmed, one probable (Figure, Table 1). The median age of cases was 65 years (interquartile range: 56─70), nine men and four women. Three individuals died (case fatality: 23%). Two cases were asymptomatic at laboratory confirmation during contact monitoring.

**Figure:**
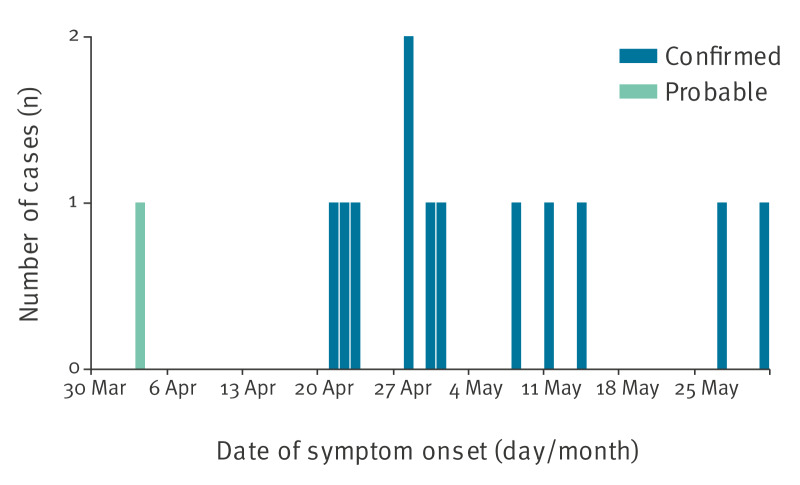
Number of human *Orthohantavirus andesense* cases by day of first illness and by case status^a^, 3 April–18 June 2026 (n = 13)

^a^ Case definitions adopted from the World Health Organization [4].

Source: Dutch National Institute for Public Health and the Environment (RIVM).

**Table 1:** Characteristics of *Orthohantavirus andesense* cases, 3 April–9 July 2026 (n = 13) ^††^

Case	Symptom onset	Case status^a^	Date of confirmation	Contact type^b^	Nationality	Age group (years)	Clinical status (as at 9 July) ^††^
1	3 Apr	Probable	Not applicable	Passenger	Dutch	60–69	Deceased 11 Apr
2	22 Apr	Confirmed	4 May	Passenger	Dutch	60–69	Deceased 26 Apr
3	21 Apr	Confirmed	2 May	Passenger	British	60–69^††^	Recovering^††^
4	23 Apr	Confirmed	8 May	Passenger	German	70–79	Deceased 2 May
5	30 Apr	Confirmed	7 May	Crew	Dutch	40–49	Recovered
6	28 Apr	Confirmed	7 May	Crew	British	50–59	Recovered
7	1 May	Confirmed	5 May	Passenger	Swiss	60–69	Recovered
8	28 Apr	Confirmed	9 Jun	Passenger	British	60–69	Recovered
9	8 May	Confirmed	11 May	Passenger	French	70–79	ICU
10	11 May	Confirmed	11 May	Passenger	Spanish	70–79	Recovered^††^
11	14 May	Confirmed	16 May^††^	Passenger	Canadian	70–79	Recovered
12	27 May	Confirmed	20 May	Crew	Dutch	40–49	Recovered
13	31 May	Confirmed	25 May	Passenger	Spanish	30–39	Recovered

ICU: intensive care unit.

^a^ Case definitions adopted from the World Health Organization [4].

^b^ All cases were passengers or crew members on board the m/v *Hondius.*

The case and contact definitions followed WHO interim guidance [4]. In brief, confirmed cases had laboratory confirmation of ANDV, probable cases were symptomatic persons with an epidemiological link with a confirmed or probable ANDV case for whom laboratory testing was not possible, and contacts were persons exposed to a probable or confirmed case while infectious. High-risk contacts included persons with shared sleeping or bathroom facilities, direct physical contact, prolonged close proximity (>15 minutes cumulative, within 2 meters) without personal protective equipment (PPE), sharing meals, prolonged social interactions, or caregiving activities; or experiencing healthcare exposure, without appropriate PPE. High-risk exposures also included handling linens, clothing, personal items, medical waste, or body fluids of a case without appropriate PPE, as well as, in the context of air travel, being seated in the same row as a case or within two rows in all directions. For the m/v *Hondius* event, all passengers and crew on board from 1 April 2026 until complete disembarkation were considered high-risk contacts, unless they consistently and appropriately used PPE throughout the exposure period.

The most likely cause of infection of the primary case (Case 1) is through zoonotic transmission from inhaling aerosolised ANDV from the urine, faeces or saliva of infected rodents. Given the symptom onset and travel history, exposure probably occurred in South America in the weeks before embarkation on the m/v *Hondius* [5]. Subsequent cases were most probably infected through person-to-person transmission on the ship, although exposure investigation continues, including potential common exposures in South America. No rodents were detected on the ship [6].

## Risk assessment

On 6 May 2026, both the ECDC and WHO assessed the risk to the general population in the lowest risk category [7,8]. Both also acknowledged the possibility of additional cases due to ANDV's potentially long incubation period of up to 42 days and possible exposure onboard the cruise ship's closed shared spaces before quarantine with daily health monitoring was implemented. Further onward transmission in (home) countries was deemed unlikely due to rapid case identification, isolation, contact tracing, quarantine and testing of high-risk contacts.

On 9 May, before final disembarkation, the ECDC classified all people onboard the cruise ship as high-risk contacts, following the precautionary principle, excluding the (public) health experts who joined the ship in Cabo Verde on 6 May and consistently used appropriate PPE. These individuals were advised to undergo 6 weeks of quarantine with active follow-up from their last potential ANDV exposure (Day 0), based on individual exposure history [9]. Upon arrival of the cruise ship in Tenerife on 10 May, disembarkation was carried out in two steps (on 10 and 12 May).

## Contact monitoring and public health response

From 13 to 15 April, the m/v *Hondius* stopped at the Tristan da Cunha islands, where one passenger (Case 8) disembarked. The ship stayed nearby for 2 nights, during which 10 local residents visited the ship; two of them stayed on board overnight. A further four residents and two travellers embarked to travel to Saint Helena. The vessel then stayed in Saint Helena from 22 to 24 April, where 32 passengers disembarked, among whom were the body of Case 1 (deceased) and Case 2 (the partner of Case 1), and one crew member embarked. Case 3 also left the ship to visit the island and reembarked later. Both Cases 2 and 3 were retrospectively identified as having experienced prodromal symptoms while ashore in Saint Helena and neither sought care at local health facilities. Extensive contact tracing in Saint Helena identified 22 high-risk contacts who entered quarantine and were followed up through active surveillance, including the six passengers who had used the vessel to transit from Tristan da Cunha. A further 67 lower-risk contacts, who either boarded the vessel for a short period or had casual contact with Cases 2 and 3, were monitored by passive surveillance. Case 2 flew to Johannesburg, South Africa, on 25 April and subsequently boarded an onward flight to the Netherlands on the same date. Before departure, Case 2 left the aircraft due to illness and died shortly thereafter. Case 3 and their partner disembarked on Ascension Island on 27 April and Case 3 was medically evacuated to South Africa on 29 April, requiring urgent care. Twelve high-risk contacts of Case 3 were later identified and followed up among the healthcare workforce on Ascension. Case 7, a Swiss resident who disembarked on Saint Helena while asymptomatic, fell ill on 1 May and was diagnosed on 4 May with ANDV after their return to Switzerland. All passengers who disembarked on Saint Helena, Tristan da Cunha or Ascension Island have been followed up. Contacts of cases, including individuals on planes, airport staff and healthcare workers have been identified and are under monitoring. As at 6 June, the 42-day monitoring period of all individuals on planes had been completed^††^, with no cases reported among passengers or personnel identified as contacts on flights. 

The ship was anchored off the coast of Praia from 3 to 6 May to facilitate the medical evacuation to the Netherlands of two symptomatic individuals (Cases 5 and 6) and a close contact of Case 4 on 6 May. This close contact was subsequently transported to Germany. On 6 May, a medical team (two physicians from the Netherlands and two public health experts from the WHO and the ECDC/European Union (EU) Health Task Force) boarded the ship in Praia (Cape Verde) to perform clinical and public health risk assessments. The ship arrived in Granadilla, Tenerife (Spain) on 10 May. At that point, 147 people were on board: 60 crew members, including expedition leaders and guides, 83 passengers and four members of the medical team. No symptomatic individuals were reported on board at that time.

On 10 May, 77 passengers and 16 crew members began disembarkation. Repatriation flights were organised to Canada, France, Ireland, mainland Spain, the Netherlands, the UK, the United States, and Türkiye, including German nationals whose transfers were coordinated via the Netherlands under public health arrangements. On 11 May, an additional 19 crew members, six passengers, the two public health experts and one physician (who was replaced by a Dutch nurse on board) were repatriated by flight to the Netherlands. There, further repatriation for non-Dutch citizens was arranged where possible, while Dutch passengers initiated their quarantine period at home. Cases 9–13 were passengers or crew repatriated on 10 or 11 May. Cases 9–11 developed symptoms after repatriation and subsequently tested positive for ANDV by laboratory testing. Cases 12 and 13 tested positive for ANDV by laboratory testing before developing symptoms.

The remaining 25 crew members and two medical personnel sailed to Rotterdam, the Netherlands, arriving on 18 May. From 18 May, five crew members remained on board to ensure the continued operation of the ship until 23 May. The Netherlands offered two quarantine facilities to those crew members who could not be immediately repatriated. On 18 June, quarantine was lifted for all 61 passengers and crew members who had been quarantined in the Netherlands, including 49 non-Dutch nationals, of which 48 are crew, quarantine has been lifted.  

In addition to ship and flight contacts, 12 healthcare and laboratory staff members at one Dutch hospital were quarantined after handling clinical waste and specimens from a confirmed case without following proper infection prevention and control measures [10]. Six of these staff members were classified as low risk on 10 June and their quarantine was lifted.

All but one of the crew members and passengers currently in quarantine in the Netherlands, as well as the remaining high-risk healthcare and laboratory contacts, have completed quarantine on 18 June. To ensure test results are available by this date, final samples have been collected 2 days before the end of the quarantine period. All PCR and serology results were negative. Additionally, for one close contact of a confirmed case, who was also on the cruise, the quarantine period was extended and completed on 2 July^††^.

As at 18 June 2026, 188 high-risk contacts were identified among the confirmed cases, representing the aggregate number of high-risk contacts placed in quarantine by the seven countries that reported cases in persons of their respective nationalities (Table 2)^††^. All these contacts are quarantined, monitored daily, and tested weekly. The last monitored contact completed quarantine on 2 July.

**Table 2:** Number of high-risk contacts of *Orthohantavirus andesense* cases, and expected quarantine completion dates by country, as at 18 June 2026 (n = 188)^a^

Country	High-risk contacts	Current latest completion of quarantine
The Netherlands	69	2 Jul^b^
United Kingdom	85	21 Jun
Canada	8	26 Jun
Germany	6	21 Jun
Switzerland	2	21 Jun
Spain	14	21 Jun
France	4	21 Jun

^a^ Update as at 2 July 2026: all quarantines for the described contacts have now been lifted, and no additional hantavirus cases have been identified among them.^††^

^b^ Latest date was extended due to being a close contact of an infected case.

On 14 May, a high-risk contact in Canada developed symptoms during follow-up and was subsequently confirmed positive for ANDV on 16 May (Case 11). On 20 May^††^, an at that time asymptomatic high-risk contact in quarantine in the Netherlands tested positive for ANDV during routine weekly screening and was moved to isolation (Case 12). Similarly, on 25 May, an asymptomatic high-risk contact in quarantine in Spain tested positive for ANDV and was transferred to isolation (Case 13).

## Diagnostics and pathogen characterisation

The ECDC and WHO have recommended immediate testing of symptomatic contacts using the ANDV-specific real-time PCR protocol developed by the EU Reference Laboratory for Public Health on Emerging, Rodent-borne, and Zoonotic Viral Pathogens (EURL-PH-ERZV), preferably on whole blood or on serum/plasma [11-13].

Andes virus RNA is most consistently detectable in blood by PCR, with less frequent and declining detection in respiratory and urinary samples over time; it can be detected in blood for up to 2 weeks before symptom onset [14]. Serological testing supports evidence of recent infection; IgM antibodies are typically detectable during the symptomatic phase, while rising IgG titres indicate recent exposure. Asymptomatic high-risk contacts are offered weekly ANDV-specific real-time PCR and serology until quarantine ends, whereas symptomatic contacts undergo immediate testing and are retested according to the ECDC and WHO testing recommendations [11,12].

The laboratory response in the EU/European Economic Area was supported by the EURL-PH-ERZV, with support from the EURL for Public Health on Vector-borne Viral Pathogens (EURL-PH-VBV), which took immediate measures to strengthen EU/EEA diagnostic capacity [13].

The preliminary sequencing analysis for five of the cases indicates a high degree of genetic similarity among sequenced cases, showing no more than one single nucleotide polymorphism difference per individual [15]. The causative ANDV was closely related to the ANDV that caused person-to-person transmission in the Epuyén, Argentina outbreak in 2018 [16]. These preliminary genomic findings support a common source or closely linked transmission chain; however, interpretation should remain cautious pending additional sequencing, epidemiological linkage and exposure data. 

The Andes virus strain related to the outbreak on the m/v *Hondius* has been isolated from a blood sample taken in the presymptomatic phase of infection of a patient who travelled on board the ship. The sample was taken as part of weekly testing in the monitoring of contacts during quarantine. The isolation confirms earlier observations of the presence of infectious ANDV in the presymptomatic phase of a clinical ANDV infection. The sample with infectious virus was taken 5 days before the onset of symptoms. With this specific sample, an ANDV-specific RT-PCR gave a positive result with a quantification cycle value of 23. The same sample was negative for both IgM and IgG against ANDV. This confirms three earlier observations were isolation of ANDV was successful in the absence of antibodies [17-19].^††^

## Discussion

Andes virus is endemic in the long-tailed pygmy rice rat (*Oligoryzomys longicaudatus*) in South America. Besides zoonotic infection, it is the only orthohantavirus with an ability for person-to-person transmission, although this requires prolonged close contact and does not appear to depend on disease severity among symptomatic cases [16,20]. Andes virus pulmonary syndrome is associated with a high case fatality, up to 50% [7,21]. The incubation period is typically 2–4 weeks, with a reported range of 1–6 weeks [16,22].

This ANDV outbreak is most consistent with one or more initial zoonotic infection(s) before embarkation, followed by secondary and possibly tertiary human-to-human transmission onboard the cruise ship in a confined setting with prolonged close contact and no visual evidence of rodents [6].

Preliminary genomic findings showing near-identical ANDV sequences in five individuals support a common source or closely linked transmission chain. The contribution of shared indoor spaces, ventilation, healthcare exposures and environmental contamination remains under investigation; environmental DNA samples collected from ship surfaces are being analysed for the presence of rodent DNA.

Several key aspects of this outbreak still need clarification: the primary transmission route for each case, whether rodent exposure or environmental contamination on board contributed to infection, which specific ship areas or activities were associated with risk, and how effectively the implemented public health measures (quarantine, contact tracing, and monitoring) prevented onward transmission in (home) countries. To address these issues, the ECDC and the National Institute for Public Health and the Environment (RIVM) in the Netherlands, in collaboration with other countries with persons in monitoring and the WHO, coordinate an epidemiological investigation of the full cohort of passengers and crew on board the m/v *Hondius* (n = 182, based on the ship company’s passengers list signed by the captain). The study collects demographics, travel history, symptoms, duties, rodent exposure, excursions and close contacts to better characterise the outbreak.

National authorities are sharing follow-up data on contacts and cases to monitor symptom onset, clinical course and outcomes. Linking clinical follow-up with longitudinal laboratory data (real-time PCR and serology) will advance understanding of ANDV infection, disease progression, transmission routes and outbreak control options. Additional data collection may include infection kinetics [23] and social/behavioural science studies to improve preparedness and response, including insights on the adherence to measures and the impact of quarantine on mental health.

## Conclusion

As at 2 July^††^, there have been 13 cases including three deaths, all among individuals who were on the ship; no cases or deaths have been reported outside of this group. No new cases have been identified as at 2 July^††^, and the quarantine period for most individuals is nearly complete. Ongoing community transmission is considered very unlikely as exposed contacts have been identified and quarantined with weekly testing and/or monitoring, and are rapidly tested if symptomatic. This outbreak highlights the need for preparedness protocols for expedition and cruise vessels, including early recognition of syndromic clusters, rapid international notification with complete information sharing on cruise ship passengers and crew and any flight contacts, as well as collaboration, implementation of onboard infection prevention and control measures, timely medical evacuation, structured risk communication, coordinated repatriation and follow-up.

## Data Availability

Outbreak investigation data such as epidemiological and serological data are available from the corresponding author upon reasonable request. Publicly available sequences analysed by Palacios et al. are available under the accession numbers PP_006WDKH.1 (Case 2), PP_006WDJK.1 (Case 3), PP_006W6RC.2 (Case 6), PP_006WBLH.2 (Case 7), PP_006W3U9.2 (Case 5) on pathoplexus.

## ^†^Author’s correction

The affiliation of Mark A Cachia was incorrect at the time of publication. This mistake was corrected on 22 June 2026 at the request of the authors.

## ^††^Addendum

On 9 July 2026, Additional information was added to this article at the request of the authors.

All quarantines for the described contacts had at that point been lifted, and no additional Andes virus cases have been identified among them. Clinical status information in Table 1 was updated indicating Case #3 as recovering, Case #9 as remaining in intensive care, and case #10 as recovered. In addition, in Table 1, the age group for Case #3 was corrected from 50–59 to 60–69 years, and the date of confirmation for Case #11 was corrected to 16 May. In the main text, the confirmation date for Case #12 was corrected from 21 May to 20 May.

The follow-up text on quarantine completion was also updated in the main article, including that the extended quarantine period for one close contact was completed on 2 July and that the 188 high-risk contacts represent the aggregate number of persons placed in quarantine by the seven countries that reported cases in persons of their respective nationalities.

Additional laboratory information was added to the Diagnostics and pathogen characterisation section stating that the Andes virus strain related to the outbreak on the m/v *Hondius* was isolated from a presymptomatic blood sample collected during weekly quarantine monitoring. The sample was obtained 5 days before symptom onset, had an ANDV-specific RT-PCR quantification cycle value of 23, and was negative for both IgM and IgG. Three references [17-19] were added in support of this information.

## UKHSA ANDV Team

Richard Puleston, Petra Manley, Grace Oswald, Will Welfare, Carol Chatt, Liam Fitzpatrick, Meera Chand, Matt Dryden, Jacob Asplin, Thomas Inns, Natalie Wright, Hilary Kirkbride, Richard Pebody, Colin Brown, Anna Aryee, Claire Gordon, Tommy Rampling, Paul Crook, Sherine Thomas.

Affiliation: UK Health Security Agency, United Kingdom.

## The International ANDV team

European Centre for Disease Prevention and Control (ECDC), Sweden: Leonidas Alexakis, Agoritsa Baka, Orlando Cenciarelli, Kostas Danis, Luna Eunkyung Park, Céline M. Gossner, Andreas Hoefer, Esther Kukielka Zunzunegui, Favelle Lamb, Emma Lof, Paula Luikens, Raffaella Melilli, Despina Pampaka, David Peres, Emmanuel Robesyn, Adriana Romaní Vidal, Gianfranco Spiteri, Giorgia Solda, Maike Winters.

British Columbia Centre for Disease Control, British Columbia, Canada: Réka Gustafson.

Sante Publique France: Alexandra Septfons, Alexandra Mailles, Arnaud Tarantola, Yvan Souares, Harold Noël.

French National, Reference Center for Hantaviruses, Institut Pasteur: Jean-Claude Manuguerra, Virginie Sauvage.

Robert Koch Institute, Berlin, Germany: Daniel Bourquain, Raskit Lachmann.

Coordinating Centre for Health Alerts and Emergencies, Ministry of Health, Madrid, Spain: Lucía García-San Miguel Rodríguez-Alarcón, Berta Suárez Rodríguez, Bernardo Guzmán Herrador, Esteban Aznar, Sonia Fernández-Balbuena, Alejandro Ciriano Cervantes , Héctor Sanchez Herrero, Juan Antonio del Castillo Polo, Gabriela Saravia Campelli, Fernando Márquez Vita, María de Salomón Arroyo, Pilar Gallego Berciano, María José Sierra Moros, Fernando Simón Soria.

General Sub-directorate of Foreign Health, Ministry of Health, Madrid, Spain: Patricia López Pereira, Rocío Palmera-Suárez, Rocío Ruiz Huertas, Beatriz Nieto Pereda, Miguel Dávila-Cornejo, Fernando Riesco Rodriguez.

General National Microbiology Center, Instituto de Salud Carlos III, Madrid, Spain: María Paz Sánchez-Seco Fariñas, Ana Isabel Negredo Antón, Ana Vázquez González.

Central de la Defensa “Gómez Ulla” Hospital, Madrid, Spain: Francisco Javier Membrillo de Novales, Tatiana Mata Forte.

General Directorate of Public Health, Generalitat Valenciana, Spain: Ana Boned-Ombuena, Maria Beso Delgado, Paula Silvestre Molines, Katja Villatoro Bongiorno.

Subdirecció General de Vigilància i Resposta a Emergències de Salut Pública, Generalitat de Catalunya, Spain: Esteve Fernández, Jacobo Mendioroz, Laura Esteban.

Centre for Infectious Disease Control, National Institute for Public Health and the Environment (RIVM), Bilthoven, The Netherlands: Núria Balanza, Olivia Luise Gern, Mart Stein, Roan Pijnacker, Annelies Brilman, Menno de Jong.

Erasmus MC-University Medical Center, Rotterdam, the Netherlands: Corine GeurtsvanKessel, Annemiek van der Eijk.

The National Institute for Communicable Diseases (NICD), South Africa: Lucille Blumberg, Nevashan Govender, Lerato Sikhosana, Jacqueline Weyer, Vuyiswa Kumalo.

World Health Organization: Emanuele Bruni, Silviu Ciobanu, Ana Paula Coutinho Rehse, Esther Hamblion, Ana Hoxha, Kareena Hundal, Zyleen Kassamali, Karen Nahapetyan, Boris I Pavlin, Ihor Perehinets, Tanja Schmidt, Marc-Alain Widdowson, Dubravka Selenic Minet.

## Note

O.E. van den Berg, N. Balanza and E. Aznar Cano and are affiliated with EPIET, and O.L. Gern is affiliated with EUPHEM. These fellowships are funded by the European Centre for Disease Prevention and Control (ECDC).
