# Author’s correction for Euro Surveill. 2026;31(24)

**DOI:** 10.2807/1560-7917.ES.2026.31.25.260625c

**Published:** 2026-06-25

**Authors:** Eurosurveillance editorial team

**Affiliations:** 1 European Centre for Disease Prevention and Control (ECDC), Stockholm, Sweden

In the article ‘Andes virus outbreak linked to expedition cruise ship travel, multi-country investigation and response, April to June 2026’ by van den Berg et al., published on 18 June 2026, the affiliation of Mark A Cachia was incorrect at the time of publication. This mistake was corrected on 22 June 2026 at the request of the authors. 

