# Addendum for Euro Surveill. 2026;31(24)

**DOI:** 10.2807/1560-7917.ES.2026.31.27.260709a

**Published:** 2026-07-09

**Authors:** Eurosurveillance editorial team

**Affiliations:** 1 European Centre for Disease Prevention and Control (ECDC), Stockholm, Sweden

Additional information was added after publication to the article ‘Andes virus outbreak linked to expedition cruise ship travel, multi-country investigation and response, April to June 2026’ by van den Berg et al., published on 18 June 2026. 

All quarantines for the described contacts had at that point been lifted, and no additional Andes virus cases have been identified among them. Clinical status information in Table 1 was updated indicating Case #3 as recovering, Case #9 as remaining in intensive care, and case #10 as recovered. In addition, in Table 1, the age group for Case #3 was corrected from 50–59 to 60–69 years, and the date of confirmation for Case #11 was corrected to 16 May. In the main text, the confirmation date for Case #12 was corrected from 21 May to 20 May.

The follow-up text on quarantine completion was also updated in the main article, including that the extended quarantine period for one close contact was completed on 2 July and that the 188 high-risk contacts represent the aggregate number of persons placed in quarantine by the seven countries that reported cases in persons of their respective nationalities. 

Additional laboratory information was added to the Diagnostics and pathogen characterisation section stating that the Andes virus strain related to the outbreak on the m/v Hondius was isolated from a presymptomatic blood sample collected during weekly quarantine monitoring. The sample was obtained 5 days before symptom onset, had an ANDV-specific RT-PCR quantification cycle value of 23, and was negative for both IgM and IgG. Three references [17-19] were added in support of this information.

These additions were made on 9 July 2026 at the request of the authors.

